# Applying Evidence-Based Medicine Principles to Hip Fracture Management

**DOI:** 10.3389/fsurg.2014.00040

**Published:** 2014-10-20

**Authors:** Joseph Bernstein, Saam Morshed, David L. Helfet, Mohit Bhandari, Jaimo Ahn

**Affiliations:** ^1^Department of Orthopaedic Surgery, University of Pennsylvania, Philadelphia, PA, USA; ^2^Department of Orthopaedic Surgery, University of California San Francisco, San Francisco, CA, USA; ^3^Department of Orthopaedic Surgery, Hospital for Special Surgery, New York, NY, USA; ^4^Department of Orthopaedic Surgery, McMaster University, Hamilton, ON, Canada

**Keywords:** fracture, orthopedic trauma, evidence-based medicine, surgical decision making, level of evidence, expert opinion, collective intelligence

## Abstract

Bone has the capacity to regenerate and not scar after injury – sometimes leaving behind no evidence at all of a prior fracture. As surgeons capable of facilitating such healing, it becomes our responsibility to help choose a treatment that minimizes functional deficits and residual symptoms. And in the case of the geriatric hip fracture, we have seen the accumulation of a vast amount of evidence to help guide us. The best method we currently have for selecting treatment plans is by the practice of evidence-based medicine. According to the now accepted hierarchy, the best is called Level I evidence (e.g., well performed randomized controlled trials) – but this evidence is best only if it is available and appropriate. Lower forms of accepted evidence include cohort studies, case control studies, case series, and case reports, and last, expert opinion – all of which can be potentially instructive. The hallmark of evidence-based treatment is not so much the reliance on evidence in general, but to use the best available evidence relative to the particular patient, the clinical setting and surgeon experience. Correctly applied, varying forms of evidence each have a role in aiding surgeons offer appropriate care for their patients – to help them best fix the fracture.

## Introduction

The management of fragility hip fractures, now 300,000 plus in the US yearly, may seem superficially to be among the most studied and settled questions in orthopedic surgery: even the geographic variations in incidence of operative management of fragility hip fractures show low variation (1). Many surgeons appear to agree with the mantra voiced in the Internet video, Orthopedics vs. Anesthesia (2): “There is a fracture; I need to fix it.”

**Figure 1:**
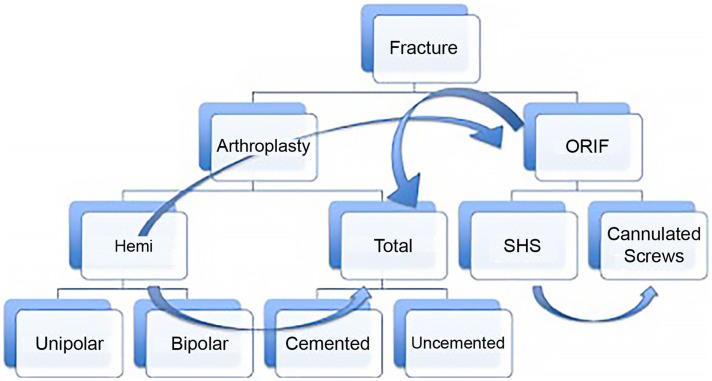
**This diagram represents the potential treatment options for a displaced femoral neck fracture with discrete nodes of decision making, which proceeds from top to bottom**. Despite attempts to make the process rational, e.g., “if fracture is displaced and the patient is elderly, consider arthroplasty instead of fixation,” other considerations may lead the surgeon from one treatment option to another (curved arrows). For instance, a 60-year-old patient with a valgus impacted fracture may initially be considered for fixation. At the other extreme, if the patient has pre-existing hip arthritis and is an active community ambulatory with minimal medical risk factors, even a non-displaced fracture may do better with total hip replacement.

Yet even if we accept that fragility fractures of the hip should be treated surgically, numerous questions remain. Which procedure is best: joint replacement or fixation? If replacement is chosen, should it be total or hemiarthroplasty? Which surgical approach is better? Should the implant be cemented? And how soon should the patient be taken to the operating room? (Figure 1). These questions are not always easy to answer for the single patient that you are confronted with. Nonetheless, they can be addressed rationally with the tools of evidence-based medicine.

The purpose of this presentation is to illustrate the methods (and limitations) of the evidence-based medicine approach in the management of fragility hip fractures.

The commonly quoted definition is attributed to Sackett (3): “Evidence-based medicine is the conscientious, explicit, and judicious use of current best evidence in making decisions about the care of individual patients. The practice of evidence-based medicine means integrating individual clinical expertise with the best available external clinical evidence from systematic research.”

This assumes that physicians’ decisions should rely on evidence of the highest quality. Accordingly “levels of evidence” hierarchies have been devised, the essence of which “is that, in general, controlled studies are better than uncontrolled studies, prospective studies are better than retrospective studies, and randomized studies are better than non-randomized studies” (4). Nevertheless, the practice of evidence-based medicine is more than simply applying the highest-level evidence: there are many reasons to not worship exclusively at the altar of randomized controlled trials. For instance, there are very few such trials in the orthopedic surgery literature. Obremskey et al. (5) reviewed 382 articles published in nine orthopedic journals and found that level I studies comprised 11.3% of the sample.

Also, randomized controlled studies can be misleading; lower level studies can be informative; expert opinion is inextricably tied to the evaluation and implementation of evidence; useful information may be found in places not even on the evidence hierarchy; perfect information about treatment options and outcomes is insufficient.

## Randomized Controlled Trials

The prospective, double-blinded randomized controlled trial is the most powerful design to avoid biases (and ultimately wrong conclusions).

- When a study is defined prospectively, the researcher will be aware of, and required to explain, subject drop out rates, lost to follow-up status, or failure of subjects to receive the treatment they were assigned to receive.
- When the study is “double blinded,” neither the subjects nor the examiner knows which treatment the subject got and thus helps avoid patients’ or surgeons’ aspirations coloring the outcomes.
- Randomization refers to the allocation of subjects to groups according to a purely chance process. A pure experiment would expose two cohorts of identical subjects to identical stimuli but for the item of interest. Because that cannot happen outside of the laboratory, the random allocation of a large number of subjects is used to ensure that our groups are as equal as possible, especially with regard to feature whose importance is unknown.
- The use of a control group allows one to state the relative efficacy of the intervention. For example, one recent study (6) reported that approximately 50% of patients whose pertrochanteric hip fracture was treated with an intramedullary nail were, at 6 months, using the same walking aids employed before they fell. In the abstract, it might be hard to say whether this is a good result or not. The contrast provided by comparator group, namely, the 40% rate in the group treated with a sliding screw, allows us to make a more meaningful inference.

Therefore, well-designed and performed randomized trials can provide the most meaningful conclusions for specific treatment questions posed. However, not all randomized controlled trials are the highest-level of evidence. Consider a recent multicenter randomized controlled trial in which reduction and fixation was compared with bipolar hemiarthroplasty and total hip replacement. In this study, 298 patients were randomized to one of the treatments. Nonetheless, this study was deemed by the Journal in which it appeared to be a level-II study, owing to the fact that the follow-up data were obtained from fewer than 80% of those enrolled. In addition, a summary statistic from a RCT may not apply to *your* patient (who may never have met inclusion criteria for the study). Larger studies that are able to stratify based on secondary independent variables of interest (e.g., active heart disease or age greater than 85) can help determine whether the conclusions apply to your particular population, though such sub-group analyses must be carefully interpreted (7). Indeed, an understanding of how similar or dissimilar your patient is to the trial population will help you properly apply the study results.

Because some trials are better than others, the best evidence is obtained from pooled analyses of trials, yet even that can come up short. Hopley et al. (8), in an effort to determine whether total hip arthroplasty was superior to hemiarthroplasty for fragility fractures of the hip, performed a systematic review and meta-analysis of four randomized controlled trials, three “quasi-randomized” trials, and eight retrospective cohort studies. Their data showed that total hip arthroplasty offers a lower risk of reoperation and better ratings in the Harris hip score at the price of slightly higher risk of dislocation and general complications, exactly the sort of information they sought. But even with pooling these 15 studies, they were forced to add that “with an overall sample size [from all of the studies] of fewer than 2000 patients our results do not allow for conclusive statements on the effectiveness of total hip arthroplasty and hemiarthroplasty for treating femoral neck fractures.”

### Mid-level evidence

By every measure, as detailed above, high-level evidence (e.g., from randomized trials) is better than mid-level evidence (e.g., from cohort studies or cases series). Nevertheless, these mid-level sources of evidence play an important role in the practice of evidence-based medicine, chiefly because high-level evidence is so rare. Brooke et al. (9), for example, in their study of trends in the quality of highly cited surgical research, found that despite improvements over the years only approximately 40% of their sample of highly cited recent papers employed high quality evidence. Beyond that, lower levels of evidence – which are more likely to highlight adverse events – can be powerful modulators of the “good news” that emanates from prospective trials. After all, as Popper noted, it takes only one black swan to refute the assertion “All swans are white.”

The lack of high quality evidence is not an indictment of researchers; sometimes mid-level evidence is best. Consider how the dangers of smoking were detected. No study took 10,000 twins, forced 5,000 of them to smoke on a regular basis for 40 years and thereafter assessed the rate of lung cancer in the two groups. Constrained by practicality, the toxic effect of cigarette smoke was found by case–control methods: studies looked at groups of people who currently have lung cancer (cases) or do not have it (controls), and assessed whether there was a higher rate of cigarette smoking in the lung cancer group. It is just not feasible to run a trial over four decades or to force a potentially detrimental “treatment” upon patients.

Ideally, evaluation of a novel surgical intervention will have a control group; and when the outcome is pain relief, the addition of a sham control group would be even better (The addition of a sham group will quantify the “placebo effect,” namely the positive change in symptoms that are related to a participant’s perceptions of their treatment rather than to the mechanisms of the treatment itself.). However, while randomization is a powerful tool in study design, it is not always feasible or ethical to proceed with a randomized, controlled trial. For example, while theory would suggest that the timing of operative debridement of an open fracture wound is best determined with a randomized trial, it is unacceptable to mandate that patients with open fractures suffer an unnecessary delay in their treatment simply because they were randomized to that treatment arm.

Observational designs can be useful tools when outcomes are rare rendering clinical trials too costly and too inefficient to answer a question in a timely manner. Most randomized trials fall victim to so-called Type II error (namely, false negative findings), simply due to an inability to recruit enough patients. On average, surgical trials are 1/10th the size needed to confirm important treatment effects.

A “differential expertise bias” is inherent in most, if not all, studies in orthopedic surgery involving surgical treatment. This is a particular concern when subjects are randomized between two groups and the skill with which they are treated may vary according to their randomization, either because the surgeon may not be equally adept with both techniques, or because different surgeons are used in the different treating arms of the study [In one study (10) comparing internal fixation, bipolar hemiarthroplasty, and total hip arthroplasty for the management of displaced subcapital fracture of the hip, consultants were twofold more likely to perform the surgery than trainees when the patient was assigned to a “replacement” treatment vs. fixation.].

### Experts and expertise

Although the levels of evidence hierarchy suggests that evidence is improved the more it is resistant to human biases, the role of experts and importance of expertise is central to the practice of evidence-based medicine. Bear in mind that clinical studies are not the findings of naturalists, collecting observations on pure biological phenomena; they are the answers to questions posed by people (researchers) recording the effects of interventions created by people (inventors) and practiced by people (surgeons) in the service of other people (patients). That is, the questions addressed in clinical papers are the questions defined by the experts; the treatments under scrutiny are the treatments devised and implemented by experts. The role of experts cannot be excised.

Furthermore, and perhaps no less importantly, clinical studies are read and employed only to guide the application of one’s individual skills, i.e., personal expertise. Consider that one study in the literature (11) has suggested that there are more dislocations of hip hemiarthroplasties inserted via the posterior approach as compared to the direct lateral route. This study of 2906 primary hemiarthroplasties found that “the overall dislocation rate for the posterior approach was 9.0% (149/1656), whereas that for the direct lateral approach was 3.3% (41/2150).” Omitting for a moment that the Cochrane review (12) on this topic stated that there is “currently insufficient evidence from randomized trials to determine the optimum surgical approach for insertion of a hemiarthroplasty to the hip,” it still is the case that at least in one specific circumstance the posterior approach is unequivocally superior: namely, when the surgeon who is asked to apply the evidence – i.e., perform the surgery – is proficient with the posterior approach and inept with the lateral. In that instance, it would be unequivocally incorrect for the surgeon to use an approach he has not mastered, regardless of what the literature has to say (The “correct” course of action, on the other hand is quite, equivocal. Should the surgeon examine her own dislocation rate and proceed if lower than 9.0%? Should the patient’s care be transferred to another surgeon who specializes in arthroplasty? How much does the patient need to know to make an informed decision? All interesting questions but beyond the scope of this manuscript.)

Overall, the literature can at best inform the reader, which of the available choices is optimal; and if what the literature suggests is not available – because of the reader’s particular expertise or lack thereof – then clearly some other solution must be applied.

The appraisal of evidence also relies on expertise. Recall that “the probability that a research finding is indeed true” (13) is not merely a function of the power of the study or the level of statistical significance of its results but also “the prior probability of it being true (before doing the study).” In that regard, a clinical study is like a diagnostic test. Just as the results of diagnostic test transform a pre-test probability of disease into a post-test probability, the results of clinical study transforms the prior truth probability of whatever the study asserts into a post study probability – the “new” truth probability.

Hence, because studies can be falsely negative or falsely positive, findings should not be accepted as canonical facts; rather, a study simply changes the likelihood that what it found is actually true. (Just think: if this assertion were wrong, there would be no need for journal clubs – what is there to discuss, if all findings are simply accepted as true?)

Consider a recent study (14) that examined 116 patients with an acute displaced femoral neck fracture. These patients were treated with either a cemented or an uncemented hemiarthroplasty and assessed for pain, mortality, mobility, and complications among other measures. Overall, the authors found that complication rates were lower in the group treated with a cemented implant and that better function and better mobility in the cemented group was suggested as well. The question remains, Should a surgeon use cement? In essence that can be translated to, “What is the probability that a research finding – that cementing is superior – is indeed true?”

The probability that cementing is superior, after considering this study, depends on two factors: how good the study was and how likely it was – before the study was done – that cementing was indeed superior. The first factor requires contemplation of potential biases and errors in the study, and the nature (level) of the evidence at hand. One must scrutinize the study with all of the tools of evidence-based medicine and make some assessment that the study as described would possibly produce false negative or false positive results. Yet the ultimate truth-value of the study depends also on the reader’s pre-test probability assessment of the results. And at some point, that prior probability is based on expert opinion – if not one’s own, than of one’s teachers, or of textbooks and the like. Medical literature cannot be interpreted without any idea of prior probability. A mere change of the prior probability from 20 to 30% determines whether the study’s findings are more likely than not to be true. And these probabilities must rest at least in part on expert opinion.

### New forms of evidence

The diagnosis of a displacement of a femoral neck fracture seems to be straightforward. Nevertheless, this is a diagnosis that is made with far from perfect accuracy. Two studies examining the accuracy of displacement of femoral neck fractures implicitly found that at least 15% of cases were mislabeled. These studies, it should be pointed out, did not set out to measure accuracy, but rather reliability. However, while reliability is not equivalent to accuracy, it is a necessary condition for it. One could not be consistently accurate without being consistent in general.

A reliability analysis regarding a binary feature (e.g., displacement) can also provide an upper bound on the average accuracy of the two viewers. That is because when two surgeons disagree about displacement, one of them must be wrong. Accordingly, each instance of disagreement represents one error (out of the two paired observations) and as such the square root of the agreement rate is an approximate upper bound on the average accuracy. Thus, when Oakes et al. (15) report an agreement level of 0.73 value, and a 0.68 value was determined by Thomsen et al. (16), the square root of these kappa values suggests an upper bound on the accuracy rate in the range of 82–85%. Given that some surgeons believe that treatment choice is driven by the presence or absence of displacement, this rate might be too high. The question is what to do about it.

One proposed approach centers on the observation that if errors are random, chance events, then greater reliability (and in turn greater accuracy) might result from having multiple readers (17). In a recent study, displacement reliability was assessed for lone readers, but also for random groups of three or five readers, with the verdict on displacement governed simply by majority rules. These groups did indeed have higher reliability: whereas the lone readers had an agreement rate of 0.69, the three and five member groups had a kappa of 0.77 and 0.80, respectively.

The amalgamation of a consensus diagnosis (now perhaps more feasible given the ubiquity of networked smart-phones and digital radiography) is a form of crowd intelligence. Crowd intelligence was first suggested by Galton (18), who noted that although butchers were more accurate than random people at guessing the weight of an ox, the mean estimate of a crowd of such ordinary people was closer to the measured weight than any individual expert’s response. (A more contemporary example is that of all of the “lifelines” employed on the TV show, who wants to be a millionaire, asking the audience for a consensus guess is the most helpful.)

Crowd intelligence can go beyond requesting diagnoses from members of a group. One hint at this new form of evidence comes from a report in *Nature* (19) that the onset of a flu epidemic might be detected by an increased frequency of searches on Google for flu remedies. As more and more patient information is stored in electronic records – and with additional information noted in registries and insurance data bases (among other sites) – it is possible that the mining these sources will produce a mother lode of worthwhile information – information that does not quite fit on the evidence hierarchy today, but may nonetheless help guide patient care.

### Beyond medical evidence

The hierarchy of medical evidence places randomized control trials at the top, but even armed with evidence from relevant randomized controlled trials one is not fully informed about which treatment option is best. That is because the medical literature provides only the list of possible treatment options for a given problem, the possible outcomes from those treatments, and the probability of reaching these outcomes. Such a collection of information, formidable though it is, is not enough to make a decision, as explained.

Medical decision making is based on a so-called expected value decision analysis. The expected value of a treatment option is the sum of the values of each possible outcome that may result from employing that option, each multiplied by the likelihood that the given outcome will be found (Values are typically expressed as “utility” points, a common currency that allows comparison between disparate entities. It need not consider monetary value at all, but it can, if financial considerations matter to those who are expressing their utilities.).

**Figure 2:**
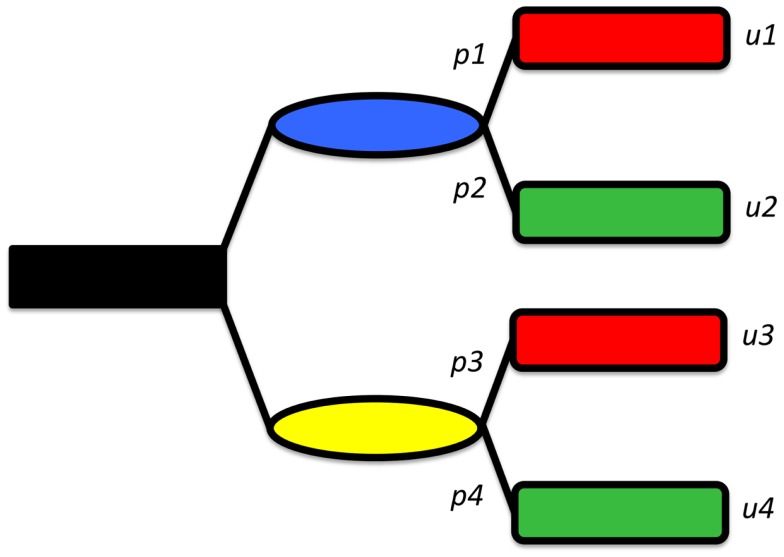
**A simplified decision tree, showing two options (shown in blue and yellow), each leading, in turn to either a “good” (green) or “bad” (red) outcome**. The probabilities of reaching each outcome are given but *p*_i_ and the values by*u*_i_.

Thus, if one knows how much each outcome is worth, and knows the likelihood that each outcome could be attained, one can measure the value of each option. Accordingly, picking the best option is simply choosing the one with the highest expected utility. Figure 2 shows a decision tree representing a very simplified synopsis of the options and possible outcomes when considering the treatment of a displaced femoral neck fracture. In this model shown, the patient is given the choice of either fracture fixation or joint replacement, each of which has (in this simplified example) one possible complication: the operation will fail and second operation will be needed. In this model, the utility value of ORIF is [(probability of success of ORIF) × (value of success of ORIF) + (probability of failure of ORIF) × (value of failure of ORIF)]. Similarly, the utility value of arthoplasty is [(probability of success of arthoplasty) × (value of success of arthoplasty) + (probability of failure of arthoplasty) × (value of failure of arthoplasty)].

With that equation in hand, one sees that even if we stipulate that the rate of revision operations is 10 times higher with fixation operations (20) (42 vs. 4%) we cannot state that fixation is the inferior choice. That is because for some particular patient, the value of a successful fixation operation may exceed the value of a successful arthroplasty by sufficient margin to make fixation preferable.

Imagine a patient assigns a utility value of 100 to successful fixation and 60 points to a successful arthroplasty (and for simplicity, we can assume that the value of the complication state is zero for both choices, allowing that term to drop out; without this assumption the math becomes a bit more complicated but the point still holds). If there is a 58% of attaining success with ORIF, then the value of this option is (100 × 0.58) + (0 × 0.42), i.e., 58 utility points. For arthroplasty, the expected utility is (60 × 0.96) + (0 × 0.04) or 57.8 points.

A surgeon practicing evidence-based medicine cannot impose his or her values on the patient, but rather must attempt (21) to discern the patient’s own values. This exercise is not easy, to be sure, as patients may not be able to express their values clearly (22), or for that matter, even hold consistent values. It may be, for example, that ORIF is deemed preferable when the patient considers its potential for success, but when the choice is framed (23) in terms of avoiding complications, a natural aversion for avoiding loss may make the arthroplasty option more appealing. To date, this dilemma – how to assess and apply patient preferences – remains unsolved.

## Concluding Remarks

The management of fragility fractures of the hip follows rule that are perhaps easy to state, but perhaps not so easily applied. We can agree that patients must be taken to the operating room as soon as possible – but how soon is that? We might agree that displaced fractures should be treated with joint replacement, but we might not agree that a given fracture is displaced. We can agree on the theoretical superiority of a particular technique, method or approach, but we also agree that a particular surgeon managing a particular case should be granted broad deference to his judgment and skills. For instance, a cemented total hip replacement inserted via the lateral approach might be most reliable, unless the surgeon is more proficient at some other technique. And we all agree that surgeons must respect patients’ autonomous decisions, even if our tools for discerning patient preferences are imperfect at best.

It may be tempting to shift the uncertainty upstream – to strive to make the best decisions to help prevent the fracture in the first place and thereby avoid the dilemmas associated with treatment. Then again, the area of prevention, too, is fraught with its own decision making dilemmas. Whitaker et al. (24) recently noted that “the optimal duration of use [of medications to prevent osteoporosis] has not been determined. Decisions to continue treatment must be based on individual assessment of risks and benefits and on patient preference. Further investigation into the benefits and risks of long-term therapy…will be crucial for determining the best regimen of treatment for individual patients with osteoporosis.”

With that in hand, we would be wise to emphasize the first clause of Sackett’s definition “The practice of evidence-based medicine means integrating individual clinical expertise with the best available external clinical evidence from systematic research.” In the end, the practice of medicine relies as much on individual clinical expertise – the art of medicine – as it does numbers, data and study conclusions. Improving the quality and quantity of clinical evidence collected – only when combined with improving the education and expertise of practitioners in applying that evidence – will lead to better surgical decision making and thereby inform us how best to fix the fracture.

## Conflict of Interest Statement

The authors declare that the research was conducted in the absence of any commercial or financial relationships that could be construed as a potential conflict of interest.
